# Enhanced terrestrial Fe(II) mobilization identified through a novel mechanism of microbially driven cave formation in Fe(III)-rich rocks

**DOI:** 10.1038/s41598-022-21365-3

**Published:** 2022-10-12

**Authors:** Ceth W. Parker, John M. Senko, Augusto S. Auler, Ira D. Sasowsky, Frederik Schulz, Tanja Woyke, Hazel A. Barton

**Affiliations:** 1 Integrated Bioscience, University of Akron, Akron, OH USA; 2 Department of Biology, University of Akron, Akron, OH USA; 3 Department of Geosciences, University of Akron, Akron, OH USA; 4 Instituto do Carste/Carste Ciência Ambiental, Belo Horizonte, MG Brazil; 5 DOE Joint Genome Institute, Lawrence Berkeley National Laboratory, Berkeley, CA USA

**Keywords:** Biogeochemistry, Biogeochemistry, Environmental microbiology

## Abstract

Most cave formation requires mass separation from a host rock in a process that operates outward from permeable pathways to create the cave void. Given the poor solubility of Fe(III) phases, such processes are insufficient to account for the significant iron formation caves (IFCs) seen in Brazilian banded iron formations (BIF) and associated rock. In this study we demonstrate that microbially-mediated reductive Fe(III) dissolution is solubilizing the poorly soluble Fe(III) phases to soluble Fe(II) in the anoxic zone behind cave walls. The resultant Fe(III)-depleted material (termed *sub muros*) is unable to maintain the structural integrity of the walls and repeated rounds of wall collapse lead to formation of the cave void in an active, measurable process. This mechanism may move significant quantities of Fe(II) into ground water and may help to explain the mechanism of BIF dissolution and REE enrichment in the generation of canga. The role of Fe(III) reducing microorganism and mass separation behind the walls (outward-in, rather than inward-out) is not only a novel mechanism of speleogenesis, but it also may identify a previously overlooked source of continental Fe that may have contributed to Archaean BIF formation.

## Introduction

The tropical regions of Brazil, including Carajás, Iron Quadrangle (IQ), and Southern Espinhaço Range, contain some of the most extensive landscapes of Proterozoic iron deposits in the world^1, 2^. The upper sequence is comprised of a laminated quartz-hematite banded iron formation, known as itabirite or jaspilite, which hosts some of the largest iron ores deposits in the world^3, 4^. The formation of these ore bodies, which represent a heterogenous mix of hematite, and goethite, and other Fe-hydroxides, is not clearly understood, but required silica removal followed by Fe replacement/deposition, with a total Fe content up to 67 wt%^2, 3, 5, 6^. These high-grade ores, which are generally low in P, Al and Si, are among the most economically important Fe deposits in the world, representing > 20% of global iron reserves^2, 7, 8^.

The iron-rich landscapes and subsurface features in Brazil are covered in a ferruginous duricrust known as canga (derived from the indigenous word itapanhoacanga^9^), which protects the more friable BIF and ore deposits from weathering^1, 2, 9–13^. This duricrust, which can range in thickness from a few centimeters to 30 m (average 3 m) is composed of detrital fragments of BIF cemented primarily by Fe-oxides, including hematite, goethite, and relatively poorly-crystalline Fe(III) (hydr)oxides^1, 9, 10, 14, 15^. This cementation generates a well indurated material surface that is extremely resistant to weathering, with rates of 0.17–0.54 m Myr^−1^ reported^10, 12, 16^. Similar to the Fe ores, the mechanism of canga formation, which includes enrichment of P oxides and rare earth elements (REEs) compared to itabirite, is poorly understood^9, 17^.

Canga does not exist as a separate bedded layer, but rather lays over the BIF landscape like a thick blanket, which led Dorr (1964) to suggest that there must be continuous turnover, otherwise it would have been removed thorough denudation long ago, exposing the friable, underlaying BIF. While canga is still much younger than BIF, it has been dated to ~ 65 Mya, making it one of the oldest exposed landscapes in Brazil^9, 10, 18^. Nonetheless, the age of canga is not homogenous, and dating has revealed that younger canga is found at depth in the same sampling cores^9, 10, 18^. In recent years, there has been increasing evidence that microbial Fe-cycling may be responsible for the maintenance of canga, with Fe(III) reduction releasing Fe(II) that is then oxidized as it moves toward the surface^9, 12, 13, 19, 20^. The abundance of consolidated Fe(III) (hydr)oxide cements at the surface make it resistant to weathering and limits water infiltration; however, discontinuities in the surface of the canga, such as tension joints, fractures and penetration by plant roots allow water to enter the subsurface, where the crust-like surface give ways to a high-porosity matrix in the canga, with an internal porosity up to 29%^1, 11, 21–23^. The routes for water into the subsurface and relatively high internal porosity of canga results in the formation of regionally significant aquifers, and water flow can reach 2.80 × 10^–4^ m s^−1^, comparable to highly fractured rocks and even karst aquifers, with primary porosity occurring at the canga-BIF interface^1, 11, 24^. Despite this porosity, the weathering-resistant nature of canga would suggest that karstification is limited; however, these iron landscapes represent some of the most cave-dense regions of Brazil, containing over 3,000 documented iron formation caves (IFCs), representing ~ 20% of all the known caves in Brazil^1, 21, 22, 25, 26^.

Brazilian IFCs were first described in 1818, and remained a relative curiosity until 1988, when the new Brazilian constitution included caves as a natural resource that required a preservation zone^1, 2, 25, 27^. This occurred during a significant increase in mining activities in Brazil, which have grown from ~ 1.5 million tons annually in the 1950s to approximately 20% of world production today (~ 400 million tons annually)^28^. Ore extraction has primarily occurred through opencast mining, and due to the preservation of identified IFCs, has necessitated a meandering pattern across the landscape, impacting ecosystems through a combination of habitat loss and the impact of mining waste effluent^27–30^.

Most IFCs are short, averaging 30 m in length with an average 2 m diameter^1^. A mechanism of formation of these IFCs was put forward by Simmons^25^, who postulated that IFCs formed due to dissolution by Fe(III) reducing microorganisms (FeRM) or through solubilization of dolomitic cements within the Itabirite. In support of this hypothesis, Parker et al.^31^ cultured FeRM from IFC sediments and demonstrated their ability to reduce Fe(III) phases within BIF and canga^31, 32^. Microbial Fe(III) reduction by cave-asssociated microorganisms was driven by fermentative organisms, which demonstrated pitting of Fe(III) (hydr)oxide surfaces^31^. While fermentation has not previously been associated with large-scale Fe-reduction, Parker et al.^31, 32^ demonstrated that it dramatically accelerated Fe-reduction when compared to respiratory Fe-reduction. Nonetheless, the reduction experiments of Parker et al.^31, 32^ were carried out in batch incubations, where passivation of Fe-oxides by Fe(II) and the closed system could reduce Fe-reduction rates and influence the drivers of FeRM metabolism^31, 33^. To better reflect the conditions experienced in canga, we demonstrated that under flow conditions bacterial fermentation Fe-reduction led to an accelerated rate of dissolution, which enhanced permeability^33^.

Together these data suggested that cave formation processes are driven by water bringing organic carbon from surficial primary productivity into the canga via the surface recharge zone through surface unconformities. Then FeRM activities reduce insoluble Fe(III) (hydr)oxides to relatively soluble Fe(II), which can then be transported via the developing aquifer^1, 26, 31, 33^. Such activity consolidates into flow paths that coalesce around cave conduits, with a mechanism of speleogenesis similar to the mass separation and transport seen in other karst systems, albeit driven by FeRM^1, 31, 34^; however, there are limitations in this model, as Fe-reduction cannot occur in the presence of oxygen (making cave formation difficult to reconcile with a conduit model) and a conduit model does not match the morphology of the observed IFCs (intercalating rooms, carved floors, no relationship with lithology)^1^. In this work, we reconcile these observations with FeRM activity using a combination of techniques in geology, materials chemistry, and geomicrobiology, to demonstrate that Fe-reduction is occurring behind the walls of the cave, sequestered from atmospheric O_2_. We demonstrate that this FeRM activity leads to extensive Fe(III)-reduction in situ, promoting passage collapse and enlargement, in an active and ongoing process that matches the observed morphology^1^. The low pH and anoxic conditions, along with the presence of apparent electron shuttles, may explain the enrichment of P oxides and REEs in canga^9^. These data demonstrate not only a novel method of cave formation, but suggest a more significant mechanism of subsurface Fe mobilization and weathering than has previously been considered^35–37^.

## Results

**Figure 1:**
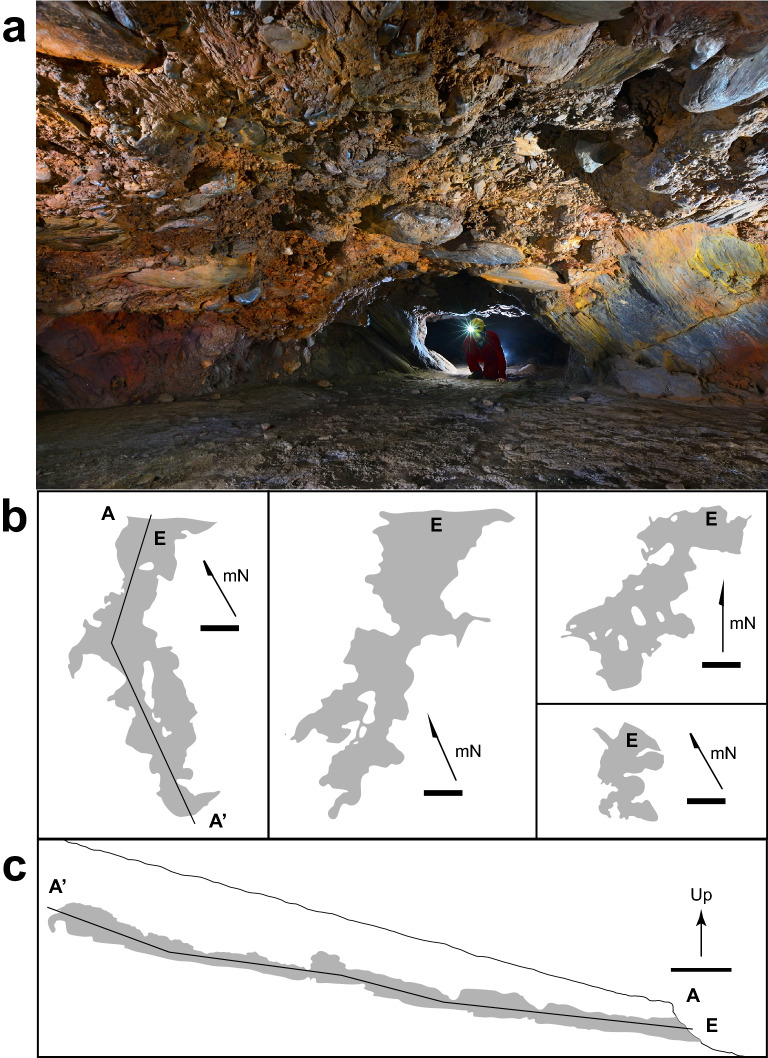
(**a**) Typical passage morphology within an IFC formed at the canga (ceiling and left wall)/BIF (right wall) interface (as described in Ref.^1^). The caver is looking into a larger chamber (ceiling ~ 2.5 m) from a smaller, connecting passage. Clastic BIF fragments embedded in canga can be seen in the ceiling. Photo courtesy of Vitor Moura and Luciana Alt. (**b**) Plan view of four IFCs demonstrating the bulbous, beads-on-a-string morphology of the cave survey. Arrows indicate magnetic North (mN), E indicates entrance, and scale bars are 5 m. The marked line (A–A’) indicates the location of the where the extended profile (shown in **c**) was recorded. (**c**) Up arrow indicates extended profile (A–A’) orientation, with the relative position of the cave to the canga surface shown. The entrance (E) and 5 m scale bar are shown.

**Figure 2:**
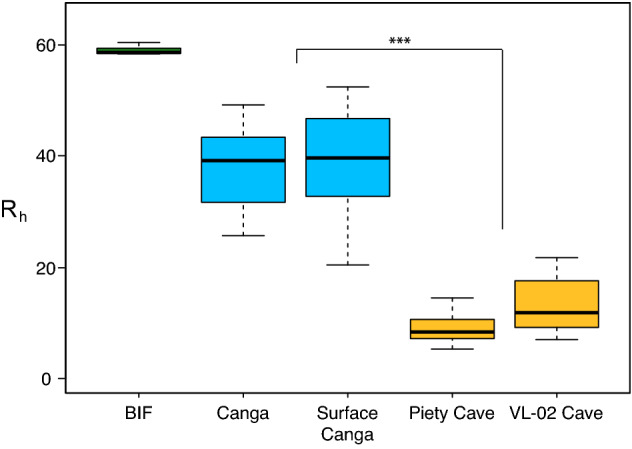
Box plot of 20 normalized Schmidt hammer rebound measurements (Rh) compared between BIF, a hand sample of canga (canga), surface canga (directly above VL-02 Cave), and walls comprised of canga in both Piety and VL-02 caves. Whiskers represent upper and lower quartiles. Significance (***) calculated using a one-way ANOVA with a P value of < 0.001.

**Figure 3:**
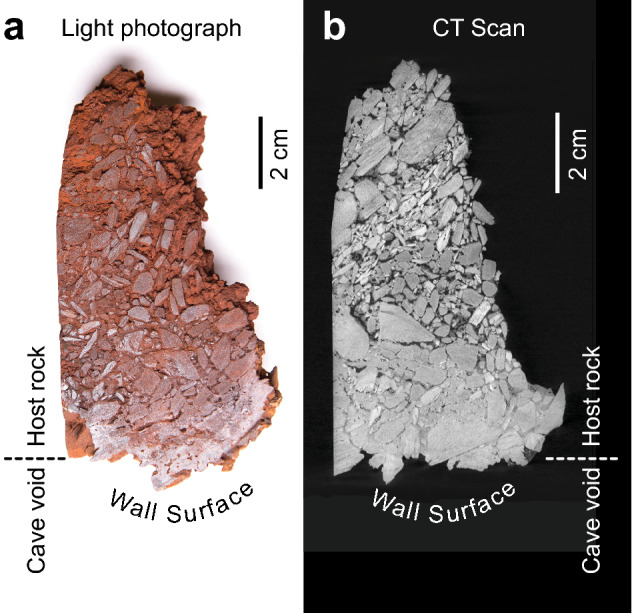
Light photography (**a**) of a hand sample taken from the wall of an IFC compared to a computed tomography (CT) slice through the same sample (**b**), with the surface oriented to the cave passage indicated. The images demonstrate the presence of BIF clasts within the canga, cementation of material on the cave face of the sample, and loss of structural goethite and increasing porosity deeper into the wall.

In our past work we characterized the microbial activity of the floor sediments of the IFCs, as this would match the traditional gravity flow path for water in epigenic caves^34^. But if dissolution was primarily occurring at floor level in these caves, this would result in a down-cutting passage morphology, which is not observed^34^. We have since quantified that the majority of IFCs (> 95%) have irregular, elliptical-shaped passageways and flat floors that align with the dip of the canga-BIF contact^1^. Given the location of the IFCs at the tops of ridges, it is untenable to invoke phreatic conditions being established to create the elliptical passage shape, and the bulbous plan of these caves (larger chambers intersected by small passageways as shown in Fig. 1) is more suggestive of wall retreat^1^. Nonetheless, if FeRM activity were driving dissolution on wall surfaces within the IFCs, we would expect there to be evidence of weathering and Fe-oxides on these surfaces; however, IFC walls are hard and smooth, with no obvious weathering. In an attempt to identify weathering zones on the cave walls we used a Schmidt hammer, which measures compressive strength of a material^38^. The data (Fig. 2) suggest that the strength of the canga walls within the caves is significantly reduced (Piety Cave, R_h_ 9.0 ± 2.4; VL-02 Cave, R_h_ 13.4 ± 4.9) compared to unaltered canga (R_h_ 38.0 ± 6.6), the canga surface directly above the cave (R_h_ 39.1 ± 9.1) and BIF (R_h_ 58.8 ± 1.0); the larger range of R_h_ values for canga are likely due to the heterogeneity of these samples manifested by the presence of hematite clasts (Fig. 3).

**Figure 4:**
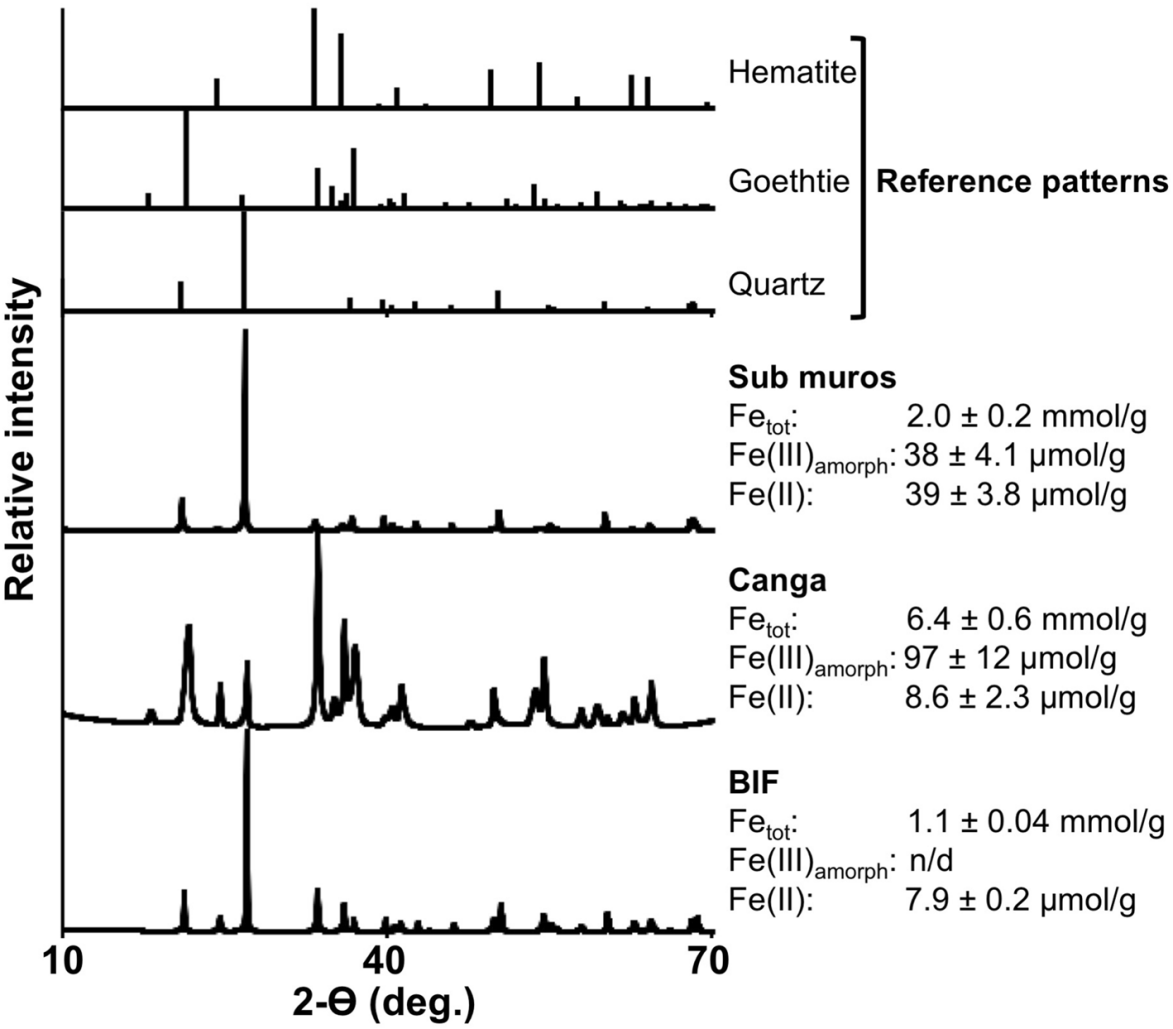
X-ray diffraction patterns and Fe content and speciation in BIF, canga, and *sub muros* associated with IFCs. Reference diffraction patterns for quartz, goethite, and hematite at the top of the figure are from Downs and Hall-Wallace, 2003. Fe_tot_, Fe(III)_amorph_, and Fe(II) represent concentrated HNO_3_/HCl-, hydroxylamine-, and 0.5 M HCl-extractable Fe (respectively).

Given that Fe(III) reduction generally requires anoxic conditions, we hypothesized that this loss of strength could be caused by Fe(III) reduction occurring behind the walls of IFCs, enhancing host rock dissolution and weakening the walls. To test this, we drilled multiple cores in a variety of locations into the walls of two separate IFCs. In all cases, the drill penetrated a hard crust, up to 3 cm thick, before entering an unconsolidated material that ranged from dry-granular to paste-like in consistency (Supplemental Fig. S1). Probing the material with a sterile spatula revealed that this unconsolidated material, which we term *sub muros* (from Latin *sub* behind + *muros* wall), extends into the canga up to 25 cm behind the cave wall surface, in all directions. A computed tomography (CT) scan of a hand sample (Fig. 3) reveals that the cave side of the wall contains cemented clasts (the hard crust we drilled through), with unconsolidated material beneath this crust. Canga is composed predominantly of goethite and hematite, while BIF contains quartz with goethite and hematite (Fig. 4). An XRD analysis of *sub muros* demonstrated that it is depleted in Fe(III) phases in comparison to the canga and BIF material (Fig. 4). Concentrated HNO_3_/HCl-extractable Fe (designated Fe_tot_ in Fig. 4) of BIF is low in comparison to other phases, and this is likely attributable to the quartz content and extreme resistance of the hematite to acid attack. Indeed, no Fe(III) could be extracted using hydroxylamine-HCl, which is used to reductively dissolve poorly crystalline Fe(III) phases (Fig. 4). Besides being relatively depleted in Fe, the *sub muros* was also enriched in Fe(II) in comparison to the other phases, indicating that Fe(III) reduction is occurring in this part of the IFCs (Fig. 4). Taken together, these results confirm that reductive dissolution of host rock Fe(III) occurs behind the cave walls, rather than at the cave wall-void interface.

### Characterization of cave-associated microorganisms

We have demonstrated enhanced porosity by FeRM activities in flow-through experiments of columns packed with crushed canga^33^, which matches the increased porosity demonstrated in the CT scan (Fig. 3). Such increased porosity would increase the capacity for meteoric water flow behind the walls and facilitate the mass export necessary for cave formation^34^. We have previously measured high levels of organic carbon in water dripping into the caves from cracks in the canga (up to 57 mg L^−1^ compared to 0.5 mg L^−1^ in carbonate caves^31, 39^), providing the carbon and energy necessary to support microbial growth and drive FeRM within the *sub muros*^31, 37^*.* When we modeled this chemistry with a synthetic pore water in vitro*,* we demonstrated significant Fe-reduction that developed a higher activity rate with repeated rounds of induced flow, presumably through FeRM selection^33^. Direct cell counting revealed ~ 3.23 × 10^7^ cells g^-1^ in the *sub muros* across the sampled caves (Supplemental Table S1), which is several orders of magnitude higher than has been seen in limestone caves (~ 10^4^–10^6^ cells g^−1^) and is just below the microbial abundance observed in soils^40, 41^.

**Figure 5:**
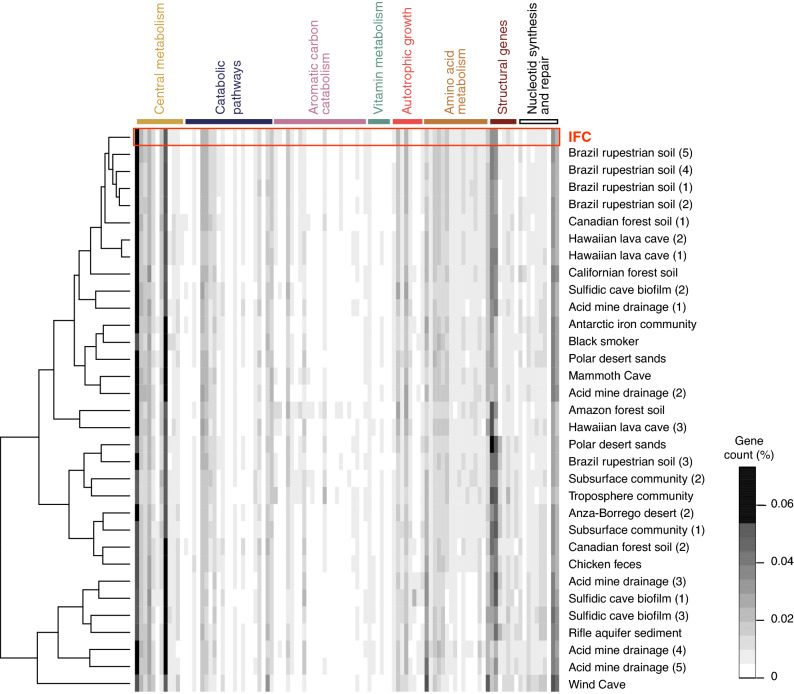
Metagenomic analysis of the microbial community within the *sub muros*, showing a comparative analysis of normalized estimated gene copy number for 103 genes identified in the KEGG functional ortholog database for 32 comparative metagenomic samples (Supplemental Table 1, with specific dataset indicated by number in paratheses). The relative abundance of each (in percentage of total gene count) is shown with a scale of none (white) up to ~ 8% (black). The dendrogram indicates community similarity determined using a hierarchical clustering function (hclustfun) in R studio. The function distribution of the identified KEGG orthologous genes is shown (detailed KEGG orthologous identification provided in Supplemental Table S1).

To determine the metabolic potential of the microorganisms found within the *sub muros,* we extracted DNA from a sample and carried out a metagenomic analysis. Illumina sequencing of *sub muric* material from Triangle Cave yielded ~ 16.78 Gbp of data for analysis. The generated dendrogram using normalized gene copy data from 103 identified KEGG orthologs genes (Fig. 5) demonstrated that the *sub muric* metabolic profile did not cluster with other microbial communities examined in other known Fe-rich systems identified in the JGI IMG database (e.g., acid mine drainage)^42, 43^. Nonetheless, the *sub muros* did share a metabolic profile with Fe-rich Brazilian rupestrian soils, similar to that expected in soils above the IFCs (Fig. 5). We observed enrichment of genes involved in amino acid and organic carbon metabolism compared to AMD and other cave environments, particularly in the breakdown of organic polymers and aromatic compounds, as would be anticipated in soil detritus (Fig. 5 and Supplemental Table S2). No photosynthetic genes were observed, confirming that the metabolic activity within the *sub muros* was isolated from photosynthetic activity, as would be expected in the aphotic environment within the cave (Fig. 5). Sequences associated with CO_2_ fixation and methane oxidation were infrequently encountered, suggesting a microbial ecosystem supported by the catabolism and assimilation of surface-derived organic carbon rather than autotrophic growth, although transcriptomics will be necessary to more clearly understand the active metabolic processes under these conditions (Fig. 5 and Supplemental Table S2).

**Figure 6:**
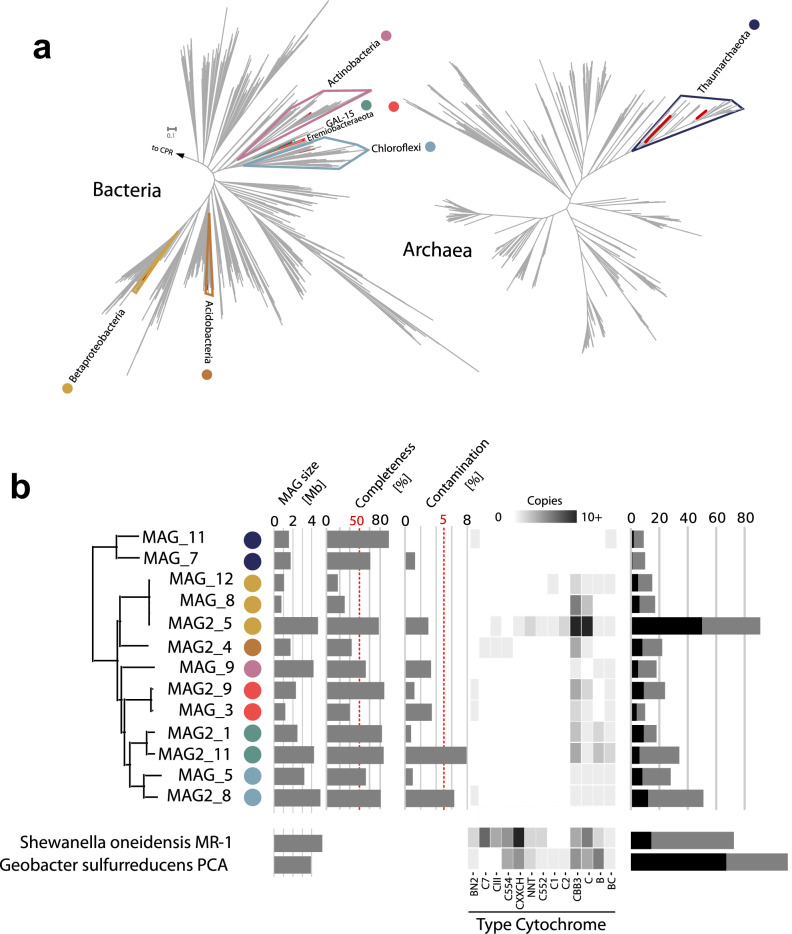
(**a**) Phylogenomic trees generated from concatenated alignments of 56 universal marker proteins. Phylogenetic position of bacterial and archaeal metagenome assembled genomes (MAGs) are highlighted with colored circles. Scale bars indicate substitutions per site. (**b**) Size, estimated completeness and contamination of cave MAGs, red dashed lines indicate cutoff for medium quality MAGs. Heatmap shows number of proteins with at least one Cytochrome domain based on PFAM annotation, counts greater than 10 are displayed as 10. Bars indicate total number of proteins with CXXCH domains (grey) and proteins with CXXCH domains and predicted transmembrane helices (black). The two genomes at the bottom represent Fe-reducing reference genomes. *Betaproteobacteria bacterium RIFCSPLOWO2_12_FULL_62_13b.

Interestingly, the metagenomic data did not demonstrate an enrichment in genes known to be associated with iron metabolism, including *mtr*ABCF and *omc*ABES^37, 44^. Nonetheless, the microbial community within the *sub muros* contained members of the *Chloroflexi*, *Acidobacteria*, WPS-2 and GAL15, which have limited cultured representatives and for which iron metabolism has not been observed or is poorly described (Fig. 6A)^37, 44^. Given the importance of *c*-type cytochromes in Fe(III) reduction, we examined the metagenomic data for the presence of the heme binding Cys-X-X-Cys-His amino acid motif. Three MAGs from the community affiliated with the *Betaproteobacteria*, the most complete of which (MAG2_5) encoded ~ 85 Cys-X-X-Cys-His motif-containing proteins, while the most complete *Chloroflexi* (MAG2_5) contained ~ 50 and the GAL15 MAG contained ~ 35 proteins with this motif (Fig. 6A). These data compare with 42 predicted *c-*type cytochromes in *Shewanella oneidensis* MR1 and 111 in *Geobacter sulfurreducens* that were identified using the same method (Fig. 6B). These findings suggest that extracellular electron transfer and dissimilatory Fe(III) reduction pathways may be present in *sub muros* communities, but involve as-yet poorly characterized genes in the microorganisms identified^45^. We also observed a relatively high abundance of genes involved in secondary metabolite production (e.g. phenazines; Supplemental Fig. S4) in the metagenomics data, which can act as electron shuttles to Fe(III) phases^46, 47^, thus providing an additional mechanism of Fe(III) reduction.

**Figure 7:**
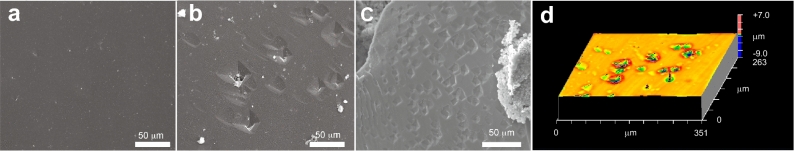
Incubated hematite coupon images. Control coupon imaged via SEM (**a**) and coupon after ~ 1 year within the cave wall (**b**). The pyramidal etch pits observed on the surface of the coupon (**b**) were similar in structure to those observed on hematite chips incubated with an IFC-derived FeRM enrichment culture (**c**)^31^. Example of optical profilometry (**d**) used to calculate the average number and volume of observed etch pits per cm^2^ in the hematite coupons (the example shown is from the same SEM image as **b**).

To determine if microbial dissolution of Fe(III) (hydr)oxides was occurring, several coupons of hematite were embedded in the *sub muros* material within the caves for approximately one year (Fig. 7). Hematite coupons were chosen because of the predominance of that phase in materials associated with the caves (Fig. 4 and Ref.^32^) and the smooth surface of the available hematite coupons which allowed us to visualize alterations of the coupon surfaces. Several attempts were made to identify microorganisms associated with the hematite chips using a number of preservation techniques (including paraformaldehyde or alcohol preservation), but none were found attached, suggesting that these organisms do not grow directly on the hematite surfaces. Instead, we observed pyramidal dissolution pits (Fig. 7B), which were identical to those seen in laboratory incubations of hematite coupons and an IFC-derived FeRM enrichment culture (Fig. 7C)^31^. Optical profilometry of the hematite coupon surface (Fig. 7D) allowed us to determine the average pit volume to be 19.7 μm^3^. This generated a total volume (*v*) loss of 5.25 × 10^4^ μ^3^ cm^−2^ of hematite surface area year^−1^. Taking into account a conservative porosity for canga (16.98%), we normalized to reactive surface area $\left( {\overline{S} } \right)$ of 148.1 cm^2^ cm^−3^^48^, allowing us to scale the in situ dissolution rate (*dS*) in μm^2^ dissolution per cm^3^ canga as:

$$dS = \frac{v}{{cm^{2} }} \times \overline{S}$$

This gave a *dS* value of 7.78 × 10^6^ μm^3^ cm^−3^. Scaling to m^3^, *dS* is therefore equivalent 7.78 cm^3^ m^−3^^32, 48^. At this rate, sufficient canga could be removed to form a typical IFC in as little as 129,032 years, although this may be an underestimation considering the faster bioreduction rates recorded for canga over hematite^32^.

## Discussion

Our goal has been to understand the processes that lead to the formation of IFCs in the relatively impermeable iron landscapes of Brazil. Caves form in many types of rock, either through erosion or dissolution^34^. The most common type of caves on Earth are epigenic caves, which form when meteoric water reacts with CO_2_ to form a weak H_2_CO_3_ solution, followed by dissolution of rock outward from the forming cave conduit and mass removal by ground water outward from the forming cave conduit^49, 50^. Microorganisms may either accelerate or play the primary role in the formation of the second most common cave type, hypogene caves^51, 52^. For example, hypogene speleogenesis in some sulfidic caves is driven by microbial metabolic activity within groundwater^52^, where aerobic oxidation of sulfide to sulfuric acid at or near the cave void-wall interface drives dissolution and cave formation^53^. In all cases, enlargement of the void occurs via dissolution outwards from the developing conduit^34^. In this “classical” model of cave formation, passage enlargement would be limited by intrusion of atmospheric O_2_ into the cave, which would limit Fe(III) reduction.

**Figure 8:**
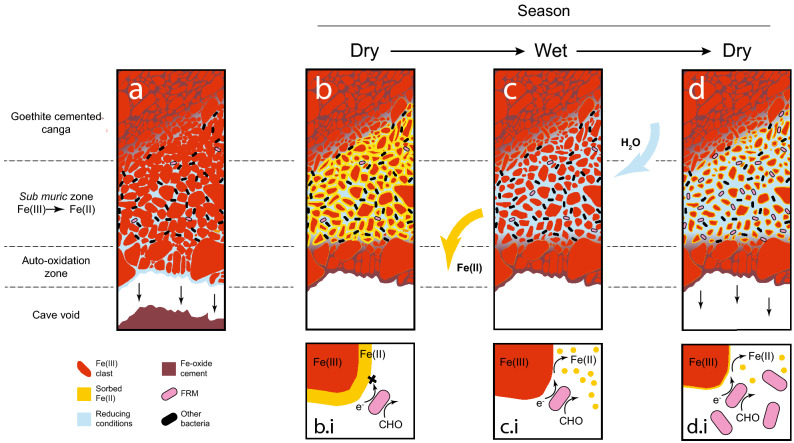
Model for FeRM-driven dissolution and speleogenesis, consolidating the data presented here and in 1, 31, and 33 (figure adapted from 1). (**a**) The *sub muros* is unable to support the Fe-oxide crust on the cave wall, which collapses into the cave and enlarges the cave void. (**b**) Fe(II) exposed to oxygen in the cave passage causes auto-oxidation, which re-forms the Fe-oxide crust on the wall surface, behind which anoxic conditions then form. At the microscopic level (**b.i)** under anoxia, Fe(III) serves as an electron acceptor leading to growth of FeRM, with Fe(II) production (as described in^31^). During the dry season, the lack of flow causes Fe(II) to passivate onto the Fe-oxides, limiting Fe-reduction (as represented by an ‘x’). (**c**) During the wet season, water enters the porous canga, introducing a pulsed flow that removes passivates (as demonstrated in Ref.^33^). These conditions (**c.i**) also bring in surface-derived organic compounds (shown as CHO) and favor Fe-reduction. (**d**) During the next dry season, the lack of pulsed flow causes the Fe(II) to begin to accumulate on surfaces again, slowing Fe-reduction and increasing FeRM abundance (**d.i**). As this cycle repeats annually, the reduction of Fe(III) increases porosity (as demonstrated in^33^), and eventually the Fe-oxide crust becomes unstable, and the collapse and Fe-reduction cycle repeats. Over time, repeated collapse leads to wall retreat and formation of the cave passages and observed morphology.

To address this paradox, we suggest a new mechanism of speleogenesis based on our results, where FeRM reductively dissolve the Fe(III)-rich matrix at the BIF/canga interface. Our previous work indicates that pulsed water flow through canga inoculated with *sub muros* enhances canga-Fe(III) reduction, at least partially due to removal of Fe(II) passivates, which would otherwise limit further Fe(III) reduction^31, 33^. This pulsed water delivery, which is consistent with rainfall patterns in the rainy season, increases FeRM activity and porosity, accelerating dissolution and the formation of Fe-depleted *sub muros* (Fig. 8)^33^. Over time, this weakening of the rock matrix causes a collapse of the Fe(III)-depleted *sub muros* material into the cave void (Fig. 8). After the collapse, oxygen within the cave atmosphere auto-oxidizes the newly exposed Fe(II)-rich fluids within the still-consolidated wall structure, cementing this matrix at the cave/wall interface (Figs. 3 and 8) and restoring the anoxic interior of the cave wall, wherein the process repeats. The mass removal of Fe(II) by water behind the wall and resultant collapse over time creates the cave void and observed cave morphology (Fig. 1)^1^. The role of Fe(III) reduction and movement of water behind the walls (outside-inwards), rather than through the cave conduit (inside-outward), is a previously undescribed mechanism of speleogenesis^34^. We term this newly recognized process *exothenic* (from the Latin *sub* behind + *muros* wall) biospeleogenesis.

Canga is enriched in P oxides, REEs, and has a positive Eu/Eu* ratio (1.8) compared to itabirite^9, 17^. It has been proposed that the enriched REEs in canga may be due to scavenging by secondary ferrihydrite oxides; however, this hypothesis does not take into account a role for microorganisms in this process^9, 33^. Given the recent disruption in the global REE supply chain, there has been a renewed interest in microbial sequestration of REEs^54^. This renewed interest has helped identify a variety of mechanisms that microorganisms utilize to immobilize REEs, including biosorption and bioaccumulation, and includes member of the Chloroflexi, Proteobacteria, Acidobacteria and Actinobacteria, that are enriched in *sub muros* (Fig. 3)^31, 33, 54, 55^. When we attempted to culture FeRM species from *sub muros,* we recovered fermentative Fe-reducing *Clostridia* spp.^33^*.* Similarly, these *Clostridia* appeared to use organic molecules to transfer electrons to Fe(III) with glucose as an electron donor with these lab-based FeRM cultures generating similar surface pitting profiles as those seen on hematite surfaces in situ (Fig. 4)^31, 33, 56^. Under the redox and pH conditions of *sub muros,* Eu would exist as Eu(III) and similar electron shuttles have been demonstrated to play a role in its reduction, which *Clostridia* bioaccumulate as intracellular polyphosphate^56, 57^. Microbial activity could therefore explain the formation of canga and relative enrichment of Eu and Nd (1.8 and 4.2 compared to itabirite, respectively)^9^.

In addition to identifying a new process of cave formation, these caves are an indication of the extensive potential for microbially mediated weathering of the rocks in the Carajás, Iron Quadrangle (IQ), and Southern Espinhaço Range, which are typically considered quite resistant to dissolutional weathering^1, 9^. Recent work^12, 13^ has indicated extensive Fe cycling in canga despite the seeming permanence of the canga. The canga is continuously reworked through alternating Fe(III) reduction and then reoxidation of the biogenic Fe(II)^12, 13^. In addition to the dynamic stability of the canga duricrust, the presence of caves and their microbial origins indicate that the itabirite phases are susceptible to substantial weathering and export of material. Here, reductively dissolved Fe may be transported through extensive subsurface aquifers, which may contribute as much as four times the freshwater discharge of rivers and streams^58, 59^. Many of these aquifers drain directly into marine environments below the surface, such as the Mediterranean Sea, which receives up to 75% its freshwater from groundwater springs that drain without detrital material that is often associated with continental runoff^60^. With a calculated dissolution value within *sub muros* of 7.78 cm^3^ m^−3^, using a density for hematite as 5.26 g cm^−3^, this would mobilize 40.9 g Fe-oxides per m^3^ canga year^−1^^61^. If this measured reductive dissolution of Fe occurred uniformly on a regional scale, assuming the canga layer is 3 m thick, this is equivalent to ~ 122 tons of Fe annually moving into the subsurface per km^2^. Given that the Iron Quadrangle alone constitutes 7200 km^2^, even if the efficiency of this system were 1% of the in situ observed values, this is equivalent to ~ 9 million tons of subsurface Fe in this region annually. As such, itabirite weathering could be an underappreciated contributor to marine Fe budgets^62, 63^. Indeed, continental Fe is proposed to be a substantial contributor to the high dissolved Fe in Archaean oceans from which BIF formed^65^.

## Materials and methods

### Field observations and sample collection

Samples were collected from three representative IFCs (Triangle Cave, CAI-03, and VL-02) within the Quadrilátero Ferrífero and Southern Espinhaco Range (MG, Brazil). Due to cave conservation practices, locations are only available upon request. The caves were < 100 m in length and averaged ~ 1.5 m in height. Due to limitations on damage, hand samples were only collected from VL-02, which was in the process of destruction through mining.

### Schmidt hammer

A Proseq L-type Schmidt hammer (Proseq, Aliquippa, PA) was used to measure rock surface rebound (R) values. The Schmidt hammer was held perpendicular to the rock surface, and the angle of orientation (θ) relative to the horizontal was recorded with a geologic compass^38^, and held constant during 20 measurements at each sampling site. The θ was used to correct for the angular effects of gravity, producing a normalized R (R_h_) value^38^. Controls were performed in a similar manner both in field tests and in a laboratory setting with samples secured to a firm iron base. R_h_ data was processed and displayed using R studio^64^. ANOVA analysis was run in Microsoft Excel for Mac Version 16.64 (Microsoft, Redmond, WA) with the StatPlus:mac Version 6 Add-In (AnalystSoft Inc, Walnut, CA).

### *Sub muros* sampling

Unless otherwise noted, all reagents were purchased from Sigma-Aldrich (St. Louis, MO). To obtain *sub muros* material we used a water-lubricated portable gasoline powered core drill with a 51 mm diameter diamond-coring bit (Shaw Tool, Yamhill, OR). The internal structure of the drill holes was unstable, and readily collapsed to expose *sub muros* that was uncontaminated by drilling fluid. While we were unable to measure pH and dissolved organic carbon of the *sub muros* used in this work, cave-associated waters (i.e. streams, dripping from speleothems, etc.) can contain ≥ 50 mg L^−1^ dissolved organic carbon and circumneutral pH, though some waters had a measured pH as low as 4.5^33^. Using aseptic technique, we collected ~ 25 mL of *sub muros* in 50 mL Falcon tubes. Samples for DNA extraction were immediately preserved in filter-sterilized 70% ethanol and stored on ice for transport, followed by storage at – 80 °C until analysis. Samples for cell counts were immediately fixed in 4% paraformaldehyde for 4 h, followed by 3× washing in phosphate buffered saline (PBS; pH 7.4). Samples for cell counting were then stored in 50% methanol/PBS at − 20 °C until counting. Samples for mineralogic analysis were collected in sterile 50 mL Falcon tubes and stored at 4 °C.

### Computed tomography

Computed tomography was carried out on a Nikon Metrology XT H 320 LC X-ray CT system (Brighton, MI) at the National Center for Education and Research on Corrosion and Materials Performance (NCERCAMP) (University of Akron, Akron, OH). The X-ray source was a 225 kV micro focus reflection target with a current of 575 µAmps and a frame capture rate of 1000 ms to produce 3,141 projections through the sample. 3D reconstruction was performed using CT-Pro Version 2.0 (Nikon Metrology, Brighton, MI). One representative hand sample was scanned, which was structurally similar to the numerous other samples collected.

### Mineralogic characterization

Samples were dried in a Thelco model 27 oven (Precision Scientific, Chicago, IL) overnight at 80 °C and pulverized in a PM 100 ball mill (Retsch, Newtown, PA). Powdered X-ray diffraction was carried out with triplicate samples on a Rigaku Ultima IV with CuKα radiation, scanning at 2Θ of 5°–70°, and an accelerating voltage of 40 kV at 35 mA. Diffraction intensities were recorded at one second intensity-counting time per step with a 0.02° step size. The resultant diffraction patterns were compared against reference diffraction patterns from the American Mineralogist Crystal Structure Database (http://rruff.geo.arizona.edu/AMS/amcsd.php) with one representative profile shown. To characterize and quantify the Fe phases within the samples, we extracted Fe(II), poorly crystalline Fe(III), and total Fe in triplicate following previously described methods^65^. Solubilization incubations for both Fe(II) and poorly crystalline Fe(III) extractions were carried out under anoxic conditions in a Coy Laboratories anaerobic chamber (Grass Lake, MI). Solubilized samples were centrifuged to remove solids and diluted in 0.5 M HCl prior to quantification via the Ferrozine assay (Stookey 1970). Total Fe extraction required an overnight digestion with 15.6 M HNO_3_ and a subsequent overnight extraction with 12.1 M HCl. Quantification was determined using atomic absorption spectrometry on a PerkinElmer AAnalyst 70 (Waltham, MA)^65^.

### In situ hematite measurements

To measure the microbial reductive activity within the IFCs, hematite coupons were prepared by adhering ~ 0.5 cm^2^ hematite coupons to 1 cm^2^ aluminum tags with silver conductive adhesive (EMS, Hatfield, PA), with ~ 15 cm of steel wire for retrieval. These coupons were wrapped in foil and sterilized in an SR24D SV autoclave at 121 °C for 30 min (Consolidated Sterilizer Systems, Boston, MA). The hematite coupons were removed from the aluminum foil within the IFCs and placed within *sub muros* through exposed boreholes, which were subsequently plugged to re-establish in situ conditions. In addition to hematite coupons in IFCs, several control coupons were left on exposed wall within the cave, along with coupons that were exposed to laboratory conditions. The coupons were incubated in place for 13 months. Upon retrieval, the coupons were immediately fixed with 4% paraformaldehyde in the field, washed, and stored as described.

For scanning electron microscopy (SEM), coupons were incubated for 1 h in a solution of 2% glutaraldehyde sodium cacodylate buffer (0.1 M at a pH of 7.2) and rinsed 3X with sodium cacodylate buffer, followed by 15 min in a solution of 4% OsO_4_ and sodium cacodylate buffer, before a final 3× washes in filter-sterilized water. The samples then underwent acetone dehydration with the use of an acetone desiccator (Fisher Scientific, Hampton, NH), followed by critical point drying in an Emitech K850 critical point drier (Emitech Ltd., Ashford, England), and sputter coated on a Polaron E5000 SEM Coating Unit (Bio-Rad, Hercules, CA) with a gold/platinum target (Bio-Rad, Hercules, CA). SEM was performed on a Hitachi S-3500 (Tarrytown, NY) with an accelerating voltage of 15.0 kV.

### Optical profilometery

To determine the extent of Fe reduction, hematite coupons were scanned on a Zygo NewView 7300 Optical Profilometer (Middlefield, CT). Etch pit identification was performed with MetroPro Version 9.1.9 software (Zygo, Middlefield, CT) using the Advance Textures 7 K applications, comparing roughness filled plots and input solid plots between the in situ IFC coupons and the in-cave control coupons. Surface roughness plots were stitched together (Supplemental Fig. S3) in Adobe Photoshop CS (Adobe, San Jose, CA), to provide a total measured area of 0.52 mm^2^ using ImageJ that was then re-sampled to determine the average number of etch pits. The pit volume was determined by averaging the volume of 15 randomly chosen etch pits, quantified using the MetroPro Microscope 7 K application (with an adjacent, artifact-free flat hematite surface used as the reference plane).

### Microbiology techniques

Direct cell counts were carried out on 100 mg of sample resuspended in PBS and stained with SYBR Green according to the manufacturers recommended protocol (Lonza, Rockland, ME). Sub-samples (100 μL) were then filtered onto a 0.2 µm GTPB Isopore Membrane Filter (Millipore, Billerica, MA). Cell counts were conducted at a 1000× magnification (frit area = 0.01 mm^2^) using a BX53 fluorescent microscope (Olympus America Inc., Center Valley, PA). Total cell numbers were determined from the average of one hundred fields-of-view as previously described^40^.

### Metagenomic analyses

Genomic DNA was extracted via the PowerMax Soil Mega Prep DNA isolation kit (Mo Bio, Carlsbad, CA) and purified on a Boreal Genomics Aurora system (Mountain View, CA) following the standard HMW DNA Soil Protocol. Metagenome sequencing was performed on the Illumina HiSeq 2500-1TB platform at the DOE JGI. The Illumina data was assembled via metaSPAdes (v3.11.1) and inferred gene content was used to calculate estimated gene copy numbers within 392 KEGG functional orthologs in the KEGG database^66^. This metagenomic data was assembled along with the metagenomic data from 32 comparative environmental sites (Supplemental Table S3), which included caves (epigenic, hypogenic and lava caves), iron-rich acid mine drainage (AMD), subsurface porewater, freshwater aquifers and iron-rich Brazilian rupestrian soils. Outlier communities for comparative purposes included forest, desert, and polar soils/sediments, a troposphere community, and chicken feces (detailed descriptions, including IMG/M pipeline accession numbers, NCBI project data and total number of bases within each metagenome can be found in Supplemental Table 1). The gene copy data across these metagenomes was normalized by dividing the estimated gene copy number per KEGG ortholog in IMG/G divided by the total estimated gene number, to provide the data to be presented as percent gene copies/genes per sequenced metagenome. There was no significant representation by eukaryotic gene counts (less than 0.01% per sample in the IFC sample). Therefore, eukaryotic orthologs were removed from analysis, leaving 120 orthologs. Of the remaining comparative orthologs, those for secondary metabolite/antibiotic synthesis demonstrated a high percentage within the samples (as has previously been associated with isolated cave environments^67^). As these data tended to dominate the heatplot analyses, they were compared separately (Supplemental Fig. S2). This left 103 orthologs for comparison. The percent gene copy heatmap was generated by first grouping the functional genes into eight metabolic categories (central metabolism, catabolic pathways, aromatic carbon catabolism, vitamin metabolism, autotrophic growth, amino acid metabolism, structural genes, and nucleotide synthesis and repair; detailed descriptions of the orthologs assigned to each group can be found in Supplemental Table S2) and using the heatmap.2 function in RStudio^64^. The gene copy percentages were used to cluster communities into similar metabolic functions using the hclustfun function, without clustering the gene orthologs (Rowv = false). The percentage gene abundance was indicated using a white/black palette using the colorRampPalette function, from no copies of the gene (white), up to 8% of the total genes within the metagenome (black). The resulting heatplot.2 output was annotated and labeled using Adobe Illustrator CS (Adobe, San Jose, CA).

### Metagenome assembly and binning.

Raw Illumina reads were trimmed, quality filtered, and corrected using bfc^68^ with the following options: -1 -s 10g -k 21 -t 10. Following quality filtering, reads were assembled with MEGAHIT and metaSPAdes (v3.11.1)^66^ with the following options: --only-assembler -k 33,55,77,99,127 --meta -t 32. The entire filtered read set was mapped to the final assembly and coverage information generated with the Burrows-Wheeler Aligner (BWA-mem^69^ and MetaBat2^70^; jgi_summarize_bam_contig_depths). Contigs were organized into genome bins with MetaBat2^70^. MAG quality in terms of completeness and contamination was determined with CheckM^71^ and overall quality determined based on the MIMAG standards^72^.

### Annotation of metagenome assembled genomes (MAGs)

Gene calling was performed with prodigal^73^ (option -meta). Cytochrome domains were identified using hmmsearch (hmmer.org, version 3.1b2) against the Pfam-A database (version 31)^74^. Proteins which contained CXXCH domains were subject to transmembrane helix prediction using the TMHMM web server (v2.0, http://www.cbs.dtu.dk/services/TMHMM/).

### Phylogenomic analysis

Medium and high-quality MAGs were added to a de-replicated set of microbial genomes available in the IMG genome taxonomy database^75^. A set of 56 universal single copy marker proteins^76^ was identified with hmmsearch using a specific hmm for each of the markers. For every marker protein, alignments were built with MAFFT (v7.294b^77^ and subsequently trimmed with BMGE using BLOSUM30^78^. Single protein alignments were then concatenated and phylogenetic trees inferred with FastTree2 using the options: -spr 4 -mlacc 2 -slownni -lg^79^.

## Supplementary Information

Supplementary Information.

## Data Availability

The metagenomic dataset is available in the NIH sequence read archive (SRA) under accession number PRJNA465920 (https://www.ncbi.nlm.nih.gov/bioproject/465920) with the annotated metagenome (Project ID Ga0187846) available through the JGI Genome Portal (https://genome.jgi.doe.gov/portal/).
