# Supplementary material for: Enhanced terrestrial Fe(II) mobilization identified through a novel mechanism of microbially driven cave formation in Fe(III)-rich rocks
Source: Sci Rep. 2022 Oct 12;12:17062. doi: 10.1038/s41598-022-21365-3 (PMC9556595; doi:10.1038/s41598-022-21365-3)
Supplement: Supplementary file 1 — Supplementary Information. [file 41598_2022_21365_MOESM1_ESM.pdf]

SUPPLEMENTAL IMAGES AND TABLES

Enhanced terrestrial Fe(II) mobilization identified through a novel mechanism of microbially driven cave formation in Fe(III)-rich rocks

Ceth W. Parker, John M. Senko, Augusto S. Auler, Ira D. Sasowsky, Federik Schulz, Tanja Woyke, and Hazel A. Barton

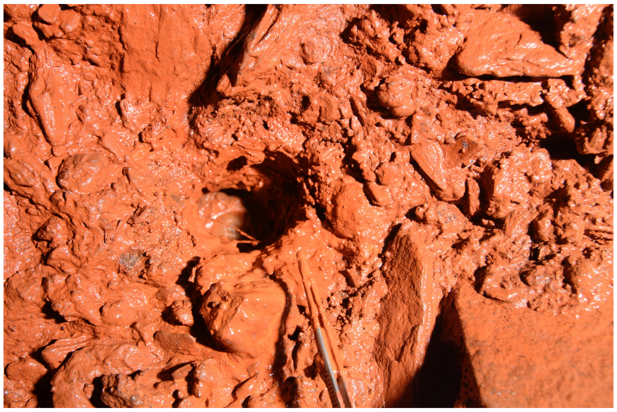

Supplemental Figure S1. The ~5 cm diameter core hole drilled into the wall of an IFC. The *sub muric* material can be seen spreading out from the hole, and on the tweezers used to probe the extent of the *sub muros*. The wire in the hole was attached to an embedded hematite chip (Figure 4), which was place in the hole before it was capped for ~1 year. This wire was then used to retrieve the hematite chip.

Supplemental Table S1. Cell counts of *sub muric* material

**Sample**	**Average cell number (cells g^{-1})**	**Standard deviation (cells g^{-1})**
BH5[a]	7.41×10^7	+/- 2.22×10^7
BH1[a]	6.61×10^6	+/- 1.52×10^6
BHB	1.63×10^7	+/- 7.94×10^6

Samples collected from CAI-03[a] and Triangle Cave[b].

Supplemental Table 2: Metabolic Grouping and Gene Order Used in Figure 5		
**Metabolic Group**	**KEGG Ortholog**	**KEGG ID**
Central Metabolism	Carbon metabolism	01200
	Glycolysis / Gluconeogenesis	00010
	Citrate cycle (TCA cycle)	00020
	Oxidative phosphorylation	00190
	Pentose phosphate pathway	00030
	Ubiquinone and other terpenoid-quinone biosyn	00130
	Fatty acid metabolism	01212
	Biosynthesis of amino acids	01230
	Lipopolysaccharide biosynthesis	00540
	Fatty acid biosynthesis	00061
	Peptidoglycan biosynthesis	00550
	Biosynthesis of unsaturated fatty acids	01040
Catabolic Pathways	Glycerolipid metabolism	00561
	Inositol phosphate metabolism	00562
	Glycerophospholipid metabolism	00564
	Ether lipid metabolism	00565
	Pyruvate metabolism	00620
	Glyoxylate and dicarboxylate metabolism	00630
	Fatty acid degradation	00071
	2-Oxocarboxylic acid metabolism	01210
	Pentose and glucuronate interconversions	00040
	Fructose and mannose metabolism	00051
	Taurine and hypotaurine metabolism	00430
	Phosphonate and phosphinate metabolism	00440
	Selenocompound metabolism	00450
	Cyanoamino acid metabolism	00460
	Galactose metabolism	00052
	D-Glutamine and D-glutamate metabolism	00471
	D-Alanine metabolism	00473
	Glutathione metabolism	00480
	Starch and sucrose metabolism	00500
	Ascorbate and aldarate metabolism	00053
	Amino sugar and nucleotide sugar metabolism	00520
	Propanoate metabolism	00640
Aromatic Carbon Metabolism	Aminobenzoate degradation	00627
	Nitrotoluene degradation	00633
	Styrene degradation	00643
	Butanoate metabolism	00650
	Biotin metabolism	00780
	Metabolism of xenobiotics by cytochrome P450	00980
	Degradation of aromatic compounds	01220
	Porphyrin and chlorophyll metabolism	00860
	Limonene and pinene degradation	00903
	Drug metabolism - cytochrome P450	00982
	Drug metabolism - other enzymes	00983
	Chlorocyclohexane and chlorobenzene degrad	00361
	Benzoate degradation	00362
	Xylene degradation	00622
	Toluene degradation	00623
	Polycyclic aromatic hydrocarbon degradation	00624

Metabolic Group	KEGG Ortholog	KEGG ID
Aromatic Carbon Metabolism	Chloroalkane and chloroalkene degradation	00625
	Naphthalene degradation	00626
	Caprolactam degradation	00930
	Cationic antimicrobial peptide (CAMP) resistance	01503
	beta-Lactam resistance	01501
	Vancomycin resistance	01502
	C5-Branched dibasic acid metabolism	00660
Vitamin Metabolism	Pantothenate and CoA biosynthesis	00770
	Thiamine metabolism	00730
	Riboflavin metabolism	00740
	Folate biosynthesis	00790
	Retinol metabolism	00830
	Vitamin B6 metabolism	00750
Autotrophic Growth	Methane metabolism	00680
	Carbon fixation in photosynthetic organisms	00710
	Carbon fixation pathways in prokaryotes	00720
	Nitrogen metabolism	00910
	Photosynthesis	00195
	Sulfur relay system	04122
	Sulfur metabolism	00920
Amino Acid Metabolism	Ribosome	03010
	Arginine biosynthesis	00220
	Alanine, aspartate and glutamate metabolism	00250
	Glycine, serine and threonine metabolism	00260
	Cysteine and methionine metabolism	00270
	Valine, leucine and isoleucine degradation	00280
	Valine, leucine and isoleucine biosynthesis	00290
	Lysine biosynthesis	00300
	Lysine degradation	00310
	Arginine and proline metabolism	00330
	Tyrosine metabolism	00350
	Phenylalanine metabolism	00360
	Tryptophan metabolism	00380
	Phenylalanine, tyrosine and tryptophan biosyn	00400
	beta-Alanine metabolism	00410
	Aminoacyl-tRNA biosynthesis	00970
Structural Genes	ABC transporters	02010
	Two-component system	02020
	Bacterial chemotaxis	02030
	Protein export	03060
	Bacterial secretion system	03070
	Cell cycle - Caulobacter	04112
	Flagellar assembly	02040
Nucleotide Synthesis and Repair	RNA degradation	03018
	RNA polymerase	03020
	DNA replication	03030
	Base excision repair	03410
	Nucleotide excision repair	03420
	Mismatch repair	03430
	Homologous recombination	03440
	Non-homologous end-joining	03450
	Purine metabolism	00230
	Pyrimidine metabolism	00240

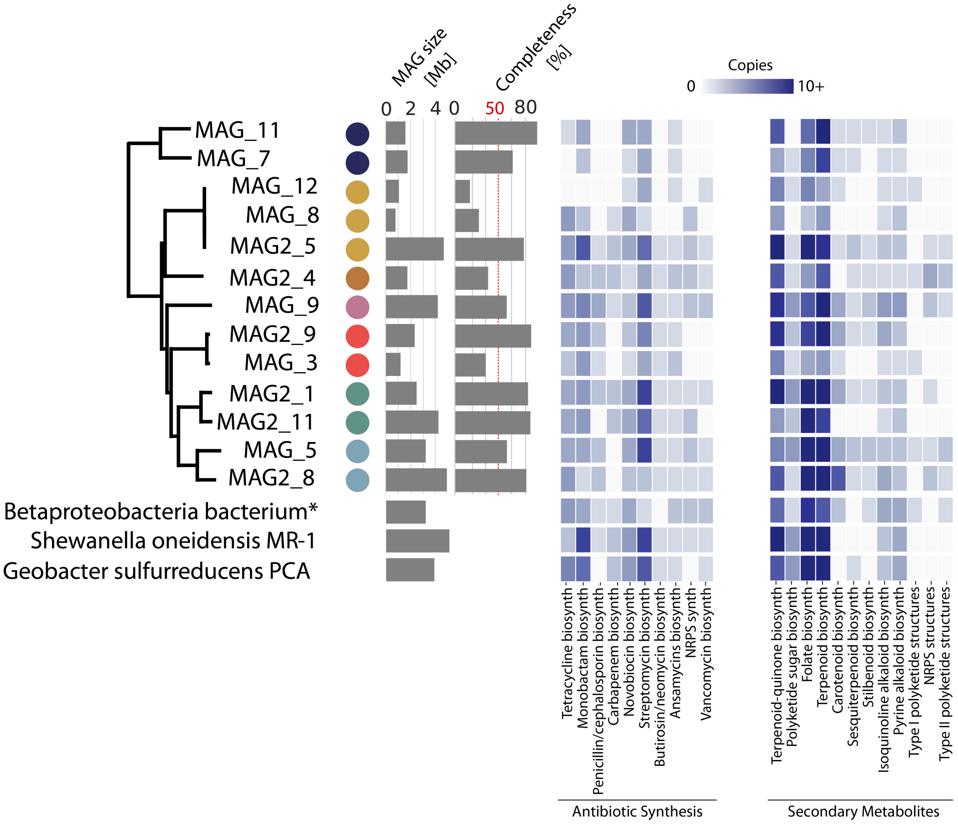

Supplemental Figure S2. Heatmap from metagenomic assembled genomes (MAGs) from the *sub muros* demonstrating the number of antibiotic biosynthesis and secondary metabolite pathways represented in each MAG. PFAM annotation counts greater than 10 are displayed as 10.

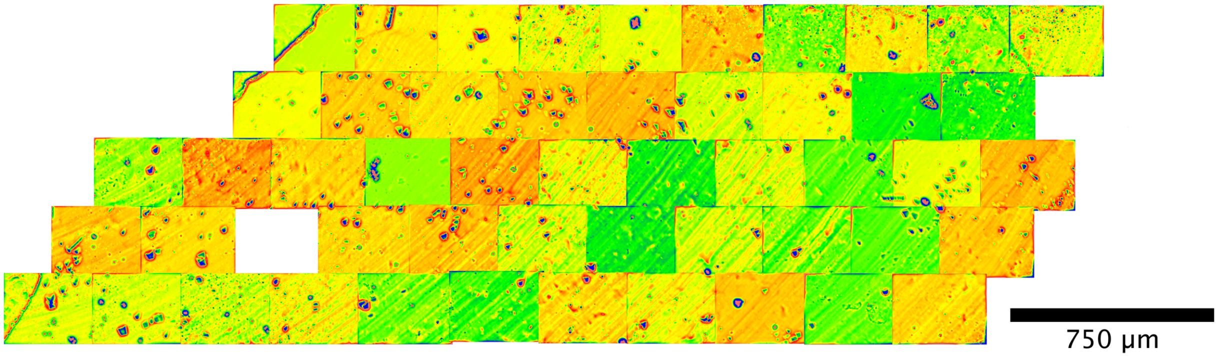

Supplemental Figure S3. Representative composite image of individual optical profilometer roughness plots of hematite coupons used to calculate etch pits/cm^2 (blue indicates depth below the surface of the hematite and red indicates height above the surface). Edge zone artifacts are due to optical interference and were removed from image analysis.

Supplemental Table S3: Metagenomic Data Used in Analyses					
**Name**	**Description**	**JGI GOLD Analysis ID***	**REF**	**# of Bases**	**Gene Count**
IFC	Biofilm microbial communities from the roof of an iron ore cave State of Minas Gerais Brazil	Ga0187846	This study	910997921	1969152
Mammoth Cave	Mammoth Cave, Echo Passage, sediment metagenome	Ga0011089		138499921	365407
Wind Cave	Freshwater microbial communities from subterranean cave lake in Wind Cave National Park	Ga0213870		527448849	1216183
Hawaiian Lava Cave (1)	Bio ooze microbial communities from a basaltic lava cave in the Kipuka Kanohina Cave System, Hawaii	Ga0187892		1260280170	2026748
Hawaiian Lava Cave (2)	White microbial mat communities from a basaltic lava cave in the Kipuka Kanohina Cave System, Hawaii	Ga0187893		1952845928	3266718
Hawaiian Lava Cave (3)	White microbial mat communities from a lava cave in the Kipuka Kanohina Cave System, Hawaii	Ga0187894		1023564476	1808083
Sulfidic Cave Biofilm (1)	Groundwater microbial communities from subsurface biofilms in sulfidic aquifier in Frasassi Gorge, Italy	Ga0026216		181303868	400347
Sulfidic Cave Biofilm (2)	Groundwater microbial communities from subsurface biofilms in sulfidic aquifier in Frasassi Gorge, Italy	Ga0026113		654101350	1509822
Sulfidic Cave Biofilm (3)	Groundwater microbial communities from subsurface biofilms in sulfidic aquifier in Frasassi Gorge, Italy	Ga0026073		448373019	974097
Subsurface community (1)	Serpentinite rock and fluid subsurface biosphere microbial communities from McLaughlin Reserve, Michigan	Ga0208243	Putman et al., 2021	158234301	255383
Subsurface community (2)	Serpentinite rock and fluid subsurface biosphere microbial communities from McLaughlin Reserve, Michigan	Ga0004656	Putman et al., 2021	125432850	253619
Rifle Aquifer Sediment	Sediment microbial communities from subsurface aquifer at Rifle CO flow through sediment column	Ga0052259	Handley et al., 2013	32714692	55879
Brazil Rupestrian Soil (1)	Barbacenia macrantha exposed rock microbial communities National Park of Serra do Cipo, Brazil	Ga0213881		1213852779	2891988
Brazil Rupestrian Soil (2)	Barbacenia macrantha exposed rock microbial communities National Park of Serra do Cipo, Brazil	Ga0213882		1237884580	3180386
Brazil Rupestrian Soil (3)	Rhizosphere microbial communities from Vellozia epidendroides National Park of Serra do Cipo, Brazil	Ga0213872		976288217	2159522
Brazil Rupestrian Soil (4)	Root associated microbial communities from Barbacenia macrantha National Park of Serra do Cipo, Brazil	Ga0213876		1620777627	3779686
Brazil Rupestrian Soil (5)	Vellozia epidendroides bulk soil microbial communities National Park of Serra do Cipo, Brazil	Ga0213878		1151465892	2802832
Canadian Forest Soil (1)	Forest soil microbial communities from Thunder Bay, Ontario, Canada	Ga0003001		29759307	94198
Canadian Forest Soil (2)	Glacier valley bacterial and archeal communities from Borup Fiord Nunavut, Canada	Ga0209724		616129211	1024020
Californian Forest Soil	Soil microbial communities from Shasta Trinity National Forest, California	Ga0233354		38554106	100962
Amazon Forest Soil	Forest soil microbial communities from Amazon forest	Ga0026787		217059300	598165
Acid Mine Drainage (1)	Sediment microbial communities from Acid Mine Drainage holding pond, Pittsburgh, PA	Ga0173609		663748475	1381741
Acid Mine Drainage (2)	Acid mine drainage microbial communities from discharge area in Wingfield Pines Conservation Area, PA	Ga0307083		36288019	42616
Acid Mine Drainage (3)	Acid mine drainage microbial communities from Los Rueldos abandoned Hg mine, Spain	Ga0052266	Méndez-García et al., 2014	14901674	24340
Acid Mine Drainage (4)	Acid Mine Drainage ARMAN microbial communities from Richmond mine, Iron Mountain, CA	Ga0074231		10830875	12568
Acid Mine Drainage (5)	Acid mine drainge microbial community from discharge pipe in Powdermill National Reserve, PA	Ga0268238		56337557	64037
Anza-Borrego Desert (1)	Soil microbial communities from Anza Borrego desert, Southern California	Ga0196970		287312560	512354
Anza-Borrego Desert (2)	Soil microbial communities from Anza Borrego desert, Southern California	Ga0196981		204709815	476996
Antarctic Iron Community	Freshwater microbial communities from Lake Bonney liftoff mats and glacier meltwater, Antarctica	Ga0105054	Tallada et al., 2022	2174974799	3504326
Polar Desert Sands	Polar desert sand microbial communities from Dry Valleys, Antarctica	Ga0136637		568254376	1279577
Troposphere Community	Upper troposphere microbial communities, Maryland	Ga0209762		49300789	89170
Black Smoker	Black smokers hydrothermal plume microbial communities from Abe Lau Basin, Pacific Ocean	Ga0012984		429147887	672341
Chicken Feces	Enriched chicken feces microbial communities from UW Madison campus, WI	Ga0160479		78306800	112814

*Visit https://gold.jgi.doe.gov/

REFERENCES:

Talladaet al. (2022). Antibiotic Resistance Genes and Taxa Analysis from Mat and Planktonic Microbiomes of Antarctic Perennial Ice-covered Lake Fryxell and Lake Bonney. *bioRxiv:* 2021.2012.2017.473166.
Putman et al., (2021) Microbial Communities in a Serpentinizing Aquifer Are Assembled through Strong Concurrent Dispersal Limitation and Selection. *mSystems* . 6(5):e0030021
Handley et al., (2013) Biostimulation induces syntrophic interactions that impact C, S and N cycling in a sediment microbial community. *ISME J.* 7(4):800-16
Méndez-García et al., (2014) Microbial stratification in low pH oxic and suboxic macroscopic growths along an acid mine drainage. *ISME J.* 8(6):1259-74
